# A little healthy competition: using mixed methods to pilot a team-based digital game for boosting medical student engagement with anatomy and histology content

**DOI:** 10.1186/s12909-015-0455-6

**Published:** 2015-10-12

**Authors:** Anna Janssen, Tim Shaw, Peter Goodyear, B. Price Kerfoot, Deborah Bryce

**Affiliations:** 1 Research in Implementation Science and e-Health Group (RISe), Faculty of Health Sciences, The University of Sydney, Sydney, NSW 2006 Australia; 2 Centre for Research on Computer Supported Learning and Cognition (CoCo), Faculty of Education and Social Work, The University of Sydney, Sydney, NSW 2006 Australia; 3 Surgical Service, Veterans Affairs Boston Healthcare System, Harvard Medical School, Boston, MA USA; 4 Discipline of Anatomy, Sydney Medical School, The University of Sydney, Sydney, NSW 2006 Australia

## Abstract

**Background:**

Digital games have been demonstrated to be beneficial for a range of non-recreational purposes, with a particular focus on their value for education. There is a limited amount of research supporting their use for medical education, but their are several studies on their use in areas such as surgical training, and life-support re-training. However, a significant gap exists in demonstrating how they engage with learners and games can be used most effectively in medical education. This pilot study assessed the value of digital games for teaching anatomy, by evaluating participant engagement and their attitudes towards a team-based strategy game.

**Methods:**

A digital game platform was designed, and then populated with anatomy questions developed by subject matter experts. Second year medical students were recruited to play three matches of the game. At the end of each match participants were asked to complete a Likert rating of their experiences of the game across five domains. Semi-structured interviews were conducted to assess engagement with the platform and perceived value to learners.

**Results:**

Sixteen participants volunteered to participate. Post-match ratings indicated that participants had a generally positive experience with the game, with 89 % of respondents agreeing the game was engaging, 93 % of respondents agreeing the game was challenging and 74 % indicating they would like to play the game again if given the opportunity.

A total of fourteen participants agreed to be interviewed after playing three matches of the game. Interview responses supported the findings of the post-match ratings that the game was considered enjoyable and engaging. Participants noted they particularly enjoyed the competitive aspect of the game, particularly the opportunity to play against peers they consider their academic equals.

In addition to finding the game engaging interview participants indicated they perceived the game impacted on their knowledge around anatomy. In particular, participants noted that the game provided them unique insight into their knowledge strengths and deficits.

**Conclusions:**

This study demonstrated that digital games can engage medical students in traditionally-challenging areas such as anatomy and offer learners unique insights into their knowledge strengths and deficits.

## Background

This paper describes a pilot study of the online team-based strategy game They Know: Anatomy. The aim of the pilot was to explore how medical students interact with the game and each other, including how they experienced the challenge, level of competitiveness, engagement, enjoyment and their desire to replay the game. It provides preliminary data on the power of games to motivate ongoing learning in a topic considered by many students to be difficult and hard to learn.

The use of digital games for non-recreational purposes is an important area of research for health educators today. It is also an area of research that has been gaining attention in the broader educational literature, with multiple studies exploring the value of digital games in areas such as developing spatial visualization skills and improving memory retention [1–3]. Much of this research has focused on demonstrating whether games can be used by educators as effective learning tools, particularly in primary and secondary education [4]. As a result there is currently a significant amount of research suggesting video games can be used productively for education: whether they can be tailored to teaching specific learning objectives, and the type of games best suited to doing so, awaits further study.

In general, the educational research community has moved away from demonstrating whether games can be used to increase knowledge, towards investigating how and why they do so [5]. However, within the health sector a significant amount of the literature continues to focus on whether digital games can improve knowledge at all. To date, the benefits of digital games has been successfully demonstrated in areas as broad as life support retraining [6], pediatric training [7], and surgical skills development [8]. A smaller portion of the literature has looked at more divergent themes such as the role of digital games in assisting mental preparedness [9]. However, further research is warranted into how to effectively utilise digital games in health education and how such tools engage learners.

In medical education core subjects like anatomy may benefit from the targeted adoption of technology-based learning tools such as digital games. Anatomy is considered a challenging subject for medical students to learn due to the complexity of the subject [10], and the breadth of the medical curriculum [11]. The literature suggests that other problems in effectively learning anatomy may include finding the topic stressful, struggling to understand the complexities of the subject and lack of confidence with the material [12, 13].

In spite of this, there appears to be no literature on the use of digital games to teach anatomy in medical education. There is one study that used a board game to encourage collaborative learning and reduce loss of confidence and concentration with medical students learning anatomy [10]. Additionally a card game was used to help reinforce content from lectures for optometry students learning anatomy [14]. Both studies were able to demonstrate playing the game resulted in a significant knowledge improvement in participants when results of a pre-test were compared to those of a post-test. Participant surveys were then used to demonstrate each games value for engaging with students and fostering collaboration amongst learners.

They Know: Anatomy is an adaption of the They Know platform for anatomy and histology. They Know is an online platform that allows educators to build team-based strategy games to support a range of curriculums. The team-based digital strategy game genre (typically) involves short-duration games in which two teams compete to meet a clear winning goal, usually over multiple matches [15]. These games are distinctive in that they cross a wide range of settings; as a result, they are able to appeal to a broad demographic of players [15]. Although research has begun to explore commercial digital games in the team-based strategy genre [16, 17], their use in medical education has not been investigated.

The focus of this study was on how players interacted with each other and how this impacted on engagement with the platform. Additionally, metrics collected by the game platform were reviewed to determine if there was an increase in the number of questions participants answered correctly across two game matches. Demonstrating a knowledge change is always important, but it is difficult to replicate the benefits of a digital game in different learning contexts without an understanding of the how and the why they are useful educational tools. It is particularly important for health educators to have information on how digital games help learners structure and enhance their knowledge, so they can successful implement them in their specific training context.

This pilot study aimed to explore how team-based digital games allowed learners to explore their existing knowledge of anatomy and histology. It also investigated how this medium was able to support team-based learning and support participant engagement with the anatomy and histology content.

## Methods

Our research study used a mixed methodology to explore the way player-player and player-platform interaction occurs in the context of an educational digital game. Our qualitative methods included self-administered participant engagement surveys, video recorded observations and semi-structured interviews. Quantitive data included metrics collected by the digital game platform on accuracy of responses to anatomy content.

### Development of the game

They Know is a platform designed for the creation of team-based strategy games in an educational setting and can be used for a variety of educational disciplines. In this study the game platform was used to develop a game, called They Know: Anatomy, to support an anatomy curriculum for medical students at The University of Sydney.

**Fig. 1:**
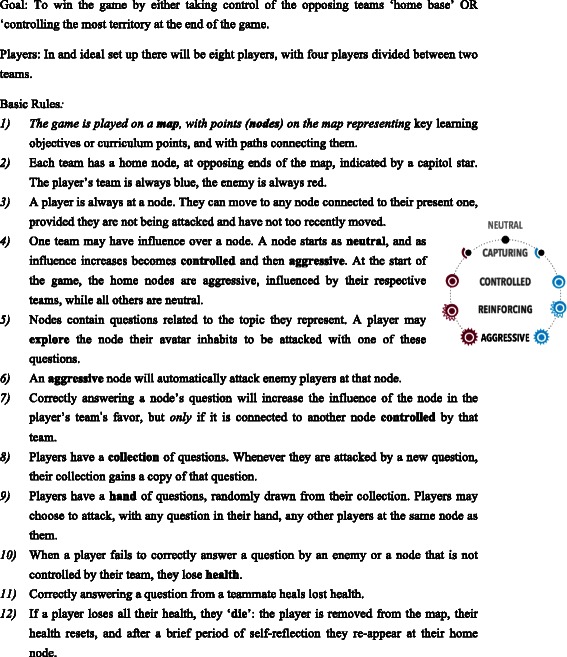
A summary of the goal of They Know, the basic rules of play and the standard number of participants per game

**Fig. 2:**
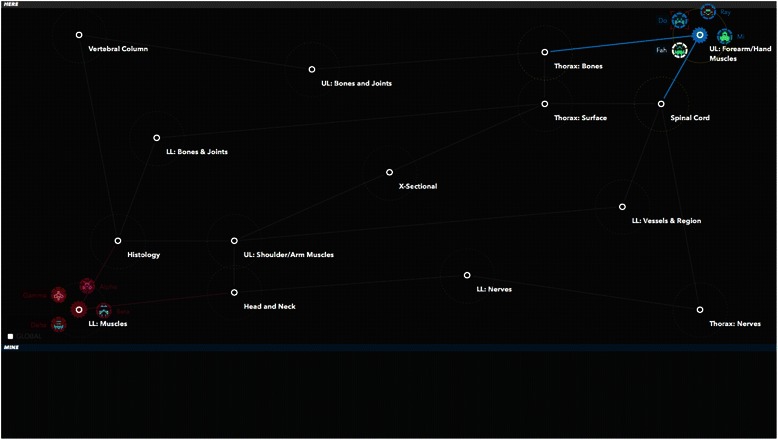
Starting screen of the game showing both teams avatars in their respective home bases. One team is represented by the blue indicator in the upper right corner, and the other as the red avatars in the lower left corner. As neither team controls any nodes on the map they all appear as white, meaning they are neutral

**Fig. 3:**
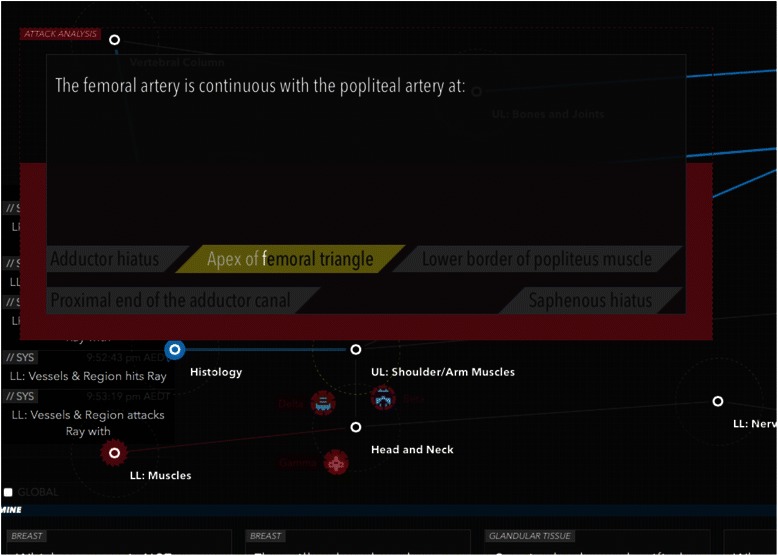
Players answer multiple choice questions on a learning objectives at each node. This figure shows the question interface for text-based questions. The red timer fills in the background whilst the player chooses a response. As they type their response the answer they have selected will begin to highlight

**Fig. 4:**
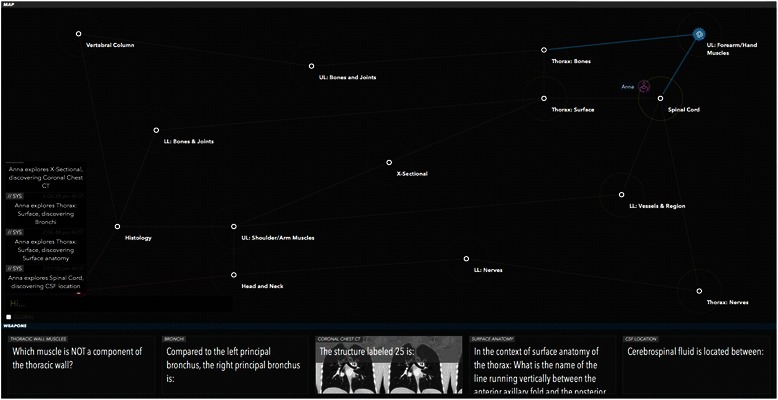
The player ‘Anna’ is exploring the Spinal Cord node. The blue lines show the line of control from the player’s home base to the node being explored. At the bottom of screen the player’s hand can be seen, made up of five random cards they have collected across from the map

The aim of any They Know game is to encourage co-operation between team mates to develop and implement a strategy to take control of the opposing teams home base. [Refer to Fig. 1 for a brief overview of game rules] [Refer to Figs. 2, 3, 4 for screenshots of the game interface] Developing a game in the platform involves distributing knowledge across a game map in a network, with each node in the network containing a set of multiple choice questions relating to a specific course objective or curriculum area.

Players start at their team’s home node, which is fully controlled by their team. When a player clicks on their current node, a question from the node’s question set is drawn randomly and posed to them. Every question has the same chance of being drawn. When a player correctly answers a question from a node connected to a node controlled by their team, the player gains more influence of their node for their team, and decreases that of their opponents. When enough influence has been gained, the node becomes controlled, turning fully blue. Players in that team can then try to take control of a neighbouring node. The first team to control their enemy’s home node wins the game.

An independent game designer developed the platform They Know over a period of twelve months. A representative of the research team met frequently with the designer to gain insight into the development process of the game platform and how it could be used effectively for developing the anatomy game. These meetings also provided opportunities for researchers to give pedagogical input into the game platform design during the development process.

Although the game can be populated with any educational topic the research team focused on anatomy. Content developers were identified from within the medical faculty at The University of Sydney to develop anatomy content for the digital game. Based on their advice, it was decided that game content would cover first year anatomy and histology knowledge in order to help students revise for their examinations in mid 2014. All content was developed, reviewed and input into the digital game They Know: Anatomy by the end of February 2014, with the aim of piloting the game by the end of March 2014. Fifteen curriculum nodes were included in the game and the content experts created ten to twenty multiple choice questions for each curriculum node. There were 240 questions in total across all nodes. [Please refer to Figs. 2, 3 and 4 for screenshots of the game].

Once the game was designed and the content had been finalized the research team decided on the placement of the curriculum nodes for the final anatomy game map. The development of this map was undertaken over several weeks and required multiple revisions to determine a layout that would encourage players to explore the maximum amount of curriculum nodes.

### Piloting the game

Second year medical students were recruited in February 2014 to participate in the study. Prior to completing a session with the game pilot participants were asked to complete a brief online survey to obtain baseline data on their level of experience with commercial video and computer games, as well as exposure and experience with educational games.

After completing the baseline survey, the participants were asked to play a game session. Each session consisted of three one-hour matches of They Know: Anatomy at spaced intervals of at least three days. Participants were allocated to play in a team of four, which remained the same across all three sessions. The matches were spaced an repeated at the specified interval to increase the likelihood of co-operation between the four player teams. The first match was designed to give players an opportunity to familiarise themselves with the rules of play and the game system. The second match was designed to increase team interaction as they players got to know each other. Finally, the third match was designed to have optimal balance of player familiarity with the game system and with team mates, resulting in collaborative team play.

During the pilot period, two game sessions consisting of three matches each were run: session A (March) and session B (August). Session A (March) was assigned a random cohort of eight participants who were then divided into two four player teams. A different group of eight participants was assigned to participate in Session B (August) and divided into two even teams as had been done during the prior session.

Participants undertook the sessions in computer labs on the University of Sydney campus, which were equipped with iMac desktop computers. Each player was given access to their own iMac computer in the computer lab which was equipped with an internet connection and a web browser, allowing participants access to the digital game which was hosted online. Each team was in the same computer lab as their other teammates but in a different computer lab to the opposing team. Players could move as individuals, controlling their own player avatar using a computer mouse to interact with nodes and access the multiple choice questions. However, in order to effectively cross the whole game map team mates had to work co-operatively and verbally communicate to coordinate their individual movements to achieve the shared goal of capturing the opposing team’s base.

### Data collection and analysis

During the matches, a video camera was set up in each of the two computer labs to record player-player interactions. Additionally screen capture software was running on each participants computer to recorded individual player-computer interaction. A session coordinator was present in both computer labs whose primary role was to provide technical support, but also to take field notes.

At the end of each match, participants were asked to complete a paper-based Likert ranking of the match. The Likert ranking asked participants to score their gameplay experience after each match across five domains: challenge, competitiveness, engagement, enjoyment, and replay likelihood. The Likert ranking ran from 1 to 6, with 6 indicating the most positive response and 1 the least positive. Scores from 1–2 were aggregated to determine the level of negative response to the game, scores from 3–4 were aggregated to determine a neutral response and scores from 5–6 were aggregated to determine positive response toward the game. Responses from all the pilot sessions were aggregated and compared across all matches in order to evaluate whether attitudes towards the domains varied across matches.

Within four weeks of completing the pilot sessions all participants were asked to participate in semi-structured interviews about their experiences with They Know: Anatomy. The semi-structured interview questions schema consisted of the following ten prompts:

1. Would you describe yourself as an experienced video game player?
2. Do you have any experience playing games you would consider educational at all? What was your view of them?
3. To what extent do you find anatomy a challenging subject?
4. How would you describe your experiences with the anatomy game you played?
5. What about this game did you find the most engaging?
6. Could you describe anything you disliked about the game?
7. How did you find the collaborative aspect of this game?
8. Did you seek advice from team mates when you were unsure how to answer a question/did you support them when they were trying to decide where to go?
9. Has playing the game impacted on how confident you feel in your understanding of anatomy?
10. Would you recommend playing the game to one of your peers?

All interviews were transcribed, de-identified, and then thematically reviewed. The review was structured to determine level of engagement participants felt playing the game and their perceived value of it as a knowledge dissemination tool.

Metrics collected by the game platform were analysed to determine the number of questions participants answered correctly across at least two matches. The number of correct answers was compared across the matches to determine a percentage change in the number or correct answers for each participant. An average was taken of the percentage change for all participants across the two matches to determine if participants increased the number of correct responses, decreased them or if response accuracy remained neutral between matches. Data was compared across only two of three matches to comply with ethics, which required participants to be given the opportunity to withdraw from the sessions early without feeling coerced. By comparing data across only two matches it was possible to comply with this requirement and still be able to use the comparison data.

Permission to conduct this study was received from the University of Sydney’s Human Research and Ethics Committee.

## Results

The demographic breakdown for the 16 participants was 13 male (81 %) and 3 female (19 %). A total of 15 participants responded to the preliminary survey with 7 (47 %) indicating they identified as experienced video game players, 4 (27 %) indicating the considered themselves occasional video game players and 4 (27 %) indicating no video game experience. In regards to exposure to educational games 6 (40 %) respondents indicated exposure to a small number of educational video games in primary and high school, and 9 (60 %) respondents did not recall ever playing an educational video game.

The first match of pilot session A (March) ran for just under two hours, so almost a whole hour over the one our time allotted, which was an unexpected outcome. When prompted by the session coordinator to end the session at the allotted time participants commented they were having so much fun playing they did not wish to end the session, noting that they wanted to wait until someone won the game. Participant reluctance to leave the session at the end of the hour was considered a demonstration of their engagement with the game system. As a result of this outcome a change was made to the system for subsequent matches: a timer function was added which declared a winner after a set time based on which team controlled the most territory. This was a significant change to the win conditions for the digital game.

In spite of the changed win conditions there was no observed decline in player engagement during subsequent pilot matches, as measured by observed verbal interactions between team mates. However, these same observations suggested participants seemed to find it less satisfying to win based on territorial control at the end of a timed period than by capturing the opposing team’s home base. Interestingly, the changed win condition did result in the players spreading out across the map more and covering more curriculum nodes, as the number of nodes they controlled would now determine if they won or lost.

At the conclusion of each match participants were asked to complete a Likert rating of various aspects of the game, with the scale using 1 to represent least agree and 6 to represent most agree. Responses from all three matches of Session A (March) and Session B (August) were combined in order to get an overall impression of how players found the game. The ranking looked at how challenging the participants found the game content, how competitive they found the game, how engaging, how enjoyable and finally their desire to play the game again.

**Fig. 5:**
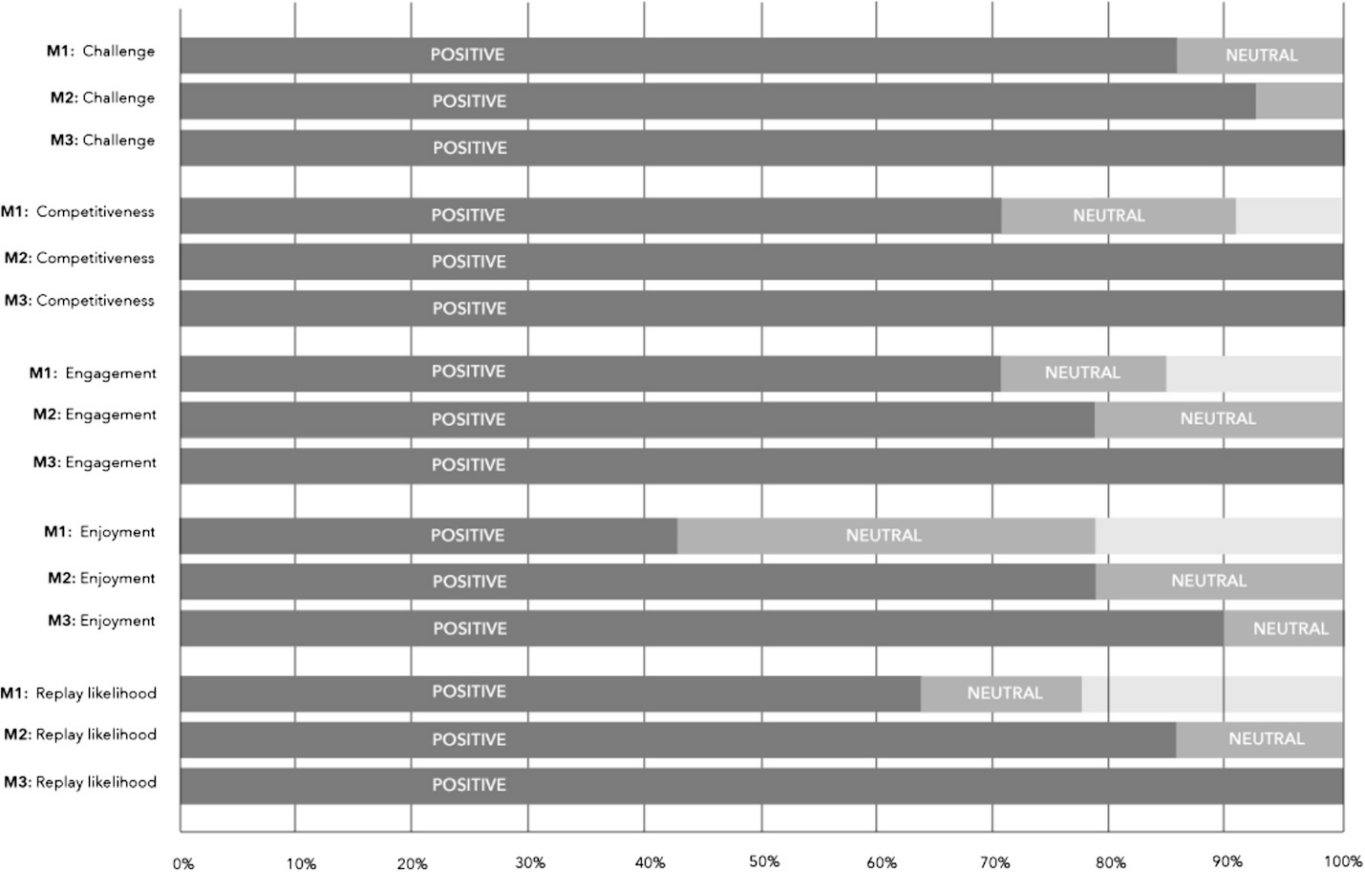
Details of the Likert scale responses compared three matches, aggregated across both sessions. Participants were asked to rate their experience of each gameplay session across the five domains shown (*n* = 16)

The vast majority of participants rated the game extremely positively, with 93 % agreeing the game was challenging, 89 % agreeing the game was competitive, 89 % agreeing the game was engaging, 73 % agreeing the game was enjoyable and 74 % indicating they would like to play the game again if given the opportunity. Although there was no comment section on the evaluation page several participants who rated the game at the lower end of enjoyment or engagement stated at the end of the match this was due to technical difficulties with the computers. Technical difficulties occurred due to the age of the computers, meaning that some computers created a ‘lagging’ effect which delayed the timers on multiple choice questions. This issue resulted in some questions being answered incorrectly due to the timer malfunctioning, but these questions were removed from the data set prior to analysis. [Please refer to Fig. 5 for visualization of Likert responses].

In addition to collecting data on how participants perceived the game, the research team also wanted to collate more objective data on the games impact on player knowledge retention across a spaced interval. In order to do this participant data collected by the game system across two matches was analyzed, to determine if there was a change in the number of questions participants answered correctly between the first and second match. When the number of questions players answered correctly across two matches was compared it was shown that, on average, players answered 11 % more questions correctly during the second match than the first. The standard deviation for this comparison was 11, with a range of −5 % to 30 %.

Three participants answered fewer questions correctly during the second match, with a decrease in accuracy of 5, 4 and 3 % in this group. However, the majority of participants (*n* = 12) showed an increase in response accuracy, with a minimum improvement of 3 % and a maximum of 30 %.

On average players visited 6.3 nodes in each match, revisiting an average of 4.8 nodes on subsequent replays. Participants encountered an average of 65 questions in a match, with a range from 20–115 questions encountered by each participant.

Data was also collected on the proportion of the match participants spent at each node. Matches were forty minutes in duration, with participants spending 2 min and 21 s of each match at a node (6 %). The minimum average time spent on a node was 39 s (1 %) and the maximum was 4 min and 33 s (11 %).

A total of 14 participants agreed to be interviewed after playing the game. Each interview was de-identified and then thematically reviewed. The final thematic analysis revealed several key themes including engagement with the game platform, using digital games as a tool for exploring the limits of knowledge and peer collaboration as vehicle for knowledge retention.

In regards to their general experiences playing the digital game, all participants indicated they found the game engaging and enjoyable. Participants particularly emphasized the impact having a challenge had in helping them engage with the game, both the challenge of playing against peers they considered their equals and the challenge level of the questions encountered in the game.

“It was fun, especially since we got to verse each other in teams. The competitive aspect of it was really entertaining… and, I think, because we all want to do well it was good motivation.”

Participants were specifically asked about the collaborative aspect of the game. Several aspects of the collaboration appealed to participants, including how the team based game created common ground for interactions with fellow students after the game. In addition players suggested that collaboration made the game more exciting and rewarding. Finally collaboration provided players with an opportunity to expand their knowledge by drawing on the knowledge of their peers to move forward in the game.

“We didn’t know certain questions and we’d consult with each other. And that came up spontaneously and it was quite fun. The sharing of knowledge… when someone helps you to clarify content, I thought that was quite fulfilling.”

Finally, participants were asked about whether they perceived playing the game impacted on their knowledge around anatomy. Almost all participants felt that playing the game impacted on their knowledge of anatomy, with several interviewees suggesting the game gave them a better understanding of their knowledge strengths and deficits. Generally participants felt that playing the game improved their confidence around the aspects of anatomy covered by the game.

“I thought it was really good in that it helped me to um, get more of a breadth of revision rather than just focusing on one thing at a time. And it was a good test as well.”

## Discussion

The results of this study suggest using a digital game can be a valuable tool to support medical student engagement with anatomy and histology content. This finding is consistent with the literature on digital games and their use as a tool for fun and engagement in education [1], as well as a valuable tool for medical education [2]. In the context of this study participants indicated they particularly enjoyed collaborating with their peers in teams. Additionally, the opportunity to compete against other medical students to win the game added an element of challenge that participants found rewarding and a motivating way to cover anatomy content. These findings build on previous research regarding the value of games to motivate medical students learning anatomy [10], by demonstrating engagement can be achieved not just with a real-world game but with a digital game.

Many studies have demonstrated digital games can disseminate knowledge as effectively as other formats and in some instances better [2, 3]. Some studies with medical students suggest that an online spaced education game can support knowledge gains [18]. In this study the data suggests that team based strategy games may help students to reinforce or increase their knowledge in the area of anatomy across two sessions. Although preliminary data from this study appears promising, the sample size of sixteen only allows us to draw preliminary conclusions. A larger study population would be required to evaluate the impact of the anatomy game on reinforcing player knowledge.

Feedback from the semi-structured interviews suggests that the game may offer learners and educators a unique means of gaining insight into the knowledge strengths and deficits of their students. This outcome was not anticipated by the research team, particularly the finding that participants found the game an unexpected tool for reflecting on the limits of their anatomy knowledge. This finding would benefit from further research into how learners might effectively integrate such a tool to enhance their revision schedules. Finally, future research is warranted into how the data collected by this style of game system could be used to impact on a curriculum.

Digital games may provide a valuable tool for educators to cost effectively integrate a novel teaching approach into their classroom to engage with learners. More research is needed to explore how digital games can be incorporated effectively into an educational curriculum, but growing data suggests they are an effective tool for engaging with learners and effectively disseminating new knowledge and will become more so in the future. Comments by participants that the digital game encouraged team work and collaboration outside of the game itself also warrants further investigation.

### Limitations

This study demonstrates that digital games can offer learners a unique means of exploring their own knowledge deficits and strengths in a timely manner. It appears that the medium is an appealing and engaging tool for many learners, especially when players can work in teams to achieve shared goals. Finally, there are traits inherent to digital games that may offer a tool for medical educators to motivate students to engage with complex curriculum areas such as anatomy.

## Conclusion

This study demonstrates that digital games may offer learners a unique means of exploring their own knowledge deficits and strengths in a timely manner. Additionally, it appears the medium is an appealing and engaging tool for many learners, especially when players can work in teams to achieve shared goals. Finally, there are traits inherent to digital games that may offer a tool for medical educators to engage students with complex curriculum areas such as anatomy.
